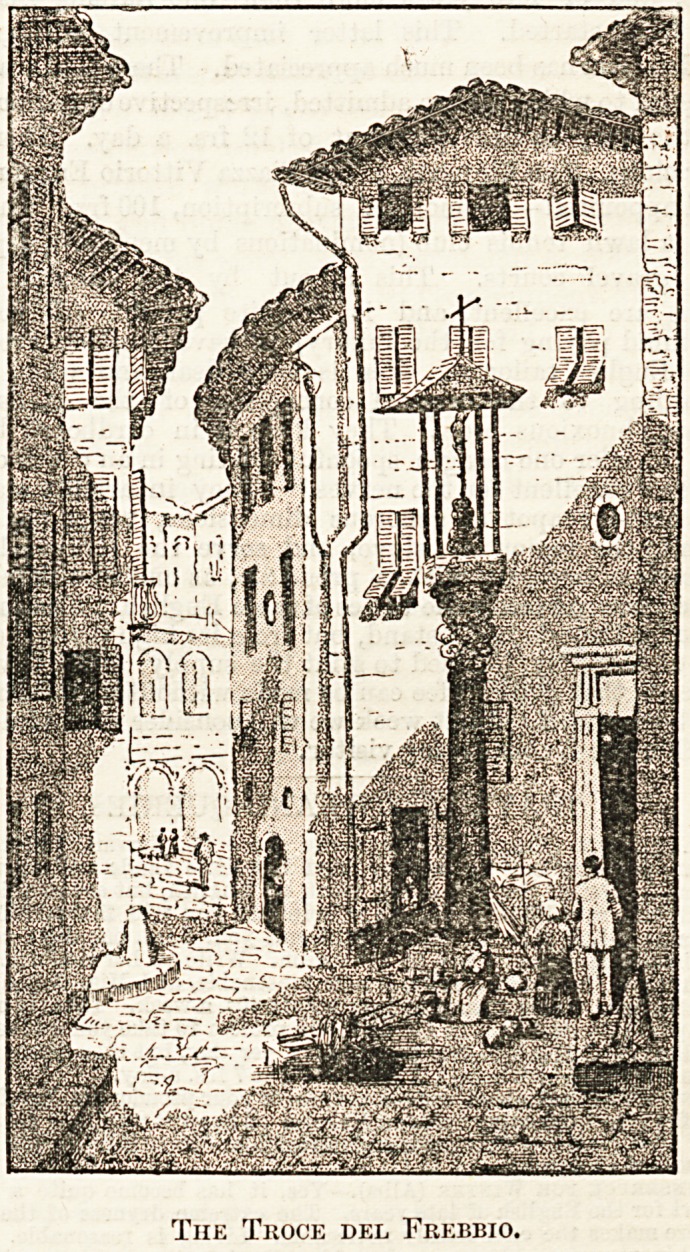# "The Hospital" Nursing Mirror

**Published:** 1899-05-27

**Authors:** 


"The Hospital" Nursing Mirror.

Being the Nursing Section of "The Hospital."

[Contributions for this Section of "The Hospital" should be addressed to the Editor, The Hospital, 28 & 29, Southampton Street, Strand, London, W.C., and should have the word "Nursing" plainly written in left-hand top corner of the envelope.]

### Notes on News from the Nursing World.

### THE AMBULANCE SCANDAL.

Once more a jury has recommended "that a trained attendant should in all cases accompany a patient conveyed in an ambulance." The question is, however, whether the recommendation will be acted upon, notwithstanding the fact that last week an omnibus driver died in an ambulance belonging to the Marylebone Guardians while he was being conveyed to the North Kensington Infirmary? It would, perhaps, be too much to say, in face of the medical evidence, that the poor man would have been alive now if the ambulance had no severely shaken him, and a nurse had been in attendance But an ambulance that shakes people is more than useless, and it is of the utmost importance that a person suffering severely should only be conveyed a long distance in charge of a trained nurse. In the case of William Salter, his wife, who accompanied him, no doubt did the best she could for him, but she was practically powerless to afford him relief. Had a nurse been in attendance she would not have started until she had known that there was some stimulant in the vehicle; and it is probable that if it had been the custom to send a nurse whenever the ambulance was used a better ambulance would have long since been provided. But we are not sanguine that the Marylebone Guardians will take the lesson to heart, unless it is brought home to them by an admonition from the Local Government Board, and a peremptory order to send out a trained nurse every time they send out an ambulance.

### THE NEW NURSES' HOME AT WHISTON.

The Prescot Board of Guardians and Whiston Rural District Council, as well as the public and the nurses themselves, are to be congratulated upon the very commodious and comfortable nurses' home which has been opened at Whiston. Built on an admirable site on the Eccleston side of the new infirmary, though separated entirely from it by the Warrington Road, the home is throughout on an adequate scale. To begin with, there are two spacious cellars embracing eight lofty large chambers, a cooking kitchen, four pantries, and two sculleries. There are three entrance-halls, two at the front and one at the back; a dining-room, 28 ft. by 20 ft.; a general assembly or twin drawing-room, 26 ft. by 24 ft. and 15½ ft. high; a library, 26 ft. by 24 ft.; a superintendent nurses' room, 18 ft. by 14 ft.; a cloak-room, two private sitting-rooms, and 31 bedrooms. Ample bathrooms and lavatory accommodation is also provided, and hot and cold water can be drawn when necessary. In case of fire there is, in addition to an internal staircase with a smoke-screen at each end of the building, an external iron staircase by which exit can be made. With praiseworthy forethought one of the internal staircases has been placed so as to serve in the event of the home requiring enlargement. At the recent opening ceremony Mr. Hallas, chairman of the Prescot Guardians, said that the proper treatment of the sick poor presupposed an efficient and a hard-working staff of nurses; and another speaker very fitly observed that "when they asked nurses to undertake the difficult duties which fell to their lot it was right that they should make all the environment of their lives as happy as they possibly could, and it was with that end in view that they had erected the nurses' home." A dance took place in the "D" block of the new infirmary to celebrate the opening.

### CONSUMPTIVE PATIENTS ON OCEAN STEAMERS.

Complaints are made that persons suffering from consumption are allowed to mix freely with other passengers on certain ocean steamers, and a case is mentioned of a vessel which left Cape Town for Plymouth lately with several who were evidently the victims of it "in a very severe form." This ought not to be, and the suggestion that a few cabins should be set aside for the exclusive use of consumptives might easily be adopted. It might, indeed, be an advantage to every-one concerned if the great steamship companies were to make a small extra charge for consumptive patients, so as to enable them to provide a trained nurse to act as stewardess to these particular passengers. Part of her duty would be to see that all due precautions were taken for the protection of the travelling public, such as the disinfection of the bedding and the cabins at the end of the journey.

### RECREATIONS IN A CHILDREN'S WARD.

It is obviously impossible for nurses, however fond of children they may be, to find time to amuse the little patients who are sufficiently convalescent to be able to play. Thrown back upon their resources, they frequently organise games for themselves, and a nurse sends an entertaining account of an incident in the children's ward of a big provincial hospital. "I had," she says, "been off duty for a short time, and on entering the ward I noticed a very strong smell of scent. Knowing that a small child had been given a small bottle, and had received permission to keep it in his locker, I asked him what had become of his scent. His answer was, 'Alfred and Ernest are playing at operlations.' I immediately went to see what the small surgeons were operating on. I found that they had an india-rubber doll with several incisions in it, which the children were very anxious to explain. 'The long one in the leg,' they said, 'was like Ada's' (periostitis); 'the knee like Ernest's' (excision of knee); 'the neck like Florrie's' (tracheotomy). They had sewn up the wounds strangely enough not over and over as one would have imagined, but each stitch with much care had been tied separately. The scent had done duty for chloroform, the corner of the sheet having been very freely sprinkled. At first I was puzzled as to what had suggested the use of the perfume, but, as the children reminded me, one of the doctors, when giving them chloroform, always told them 'to smell the nice scent, like mother puts on her handkerchief.'"

### A BANKRUPT NURSE.

Many nurses are bitten with the idea that if they could only start a nursing home they would be prosperous and happy. It is rarely that any of the numerous failures are heard of, therefore the history of Miss Amy Florence Fry, which was told to the Official Receiver of Bradford, is especially instructive. She trained as nurse in 1891, and in 1897 she and a friend organised the West Riding Nurses' Association at Halifax. This venture was unsuccessful, and she next established a private home hospital at Bradford. The objections of her neighbours to a hospital in their midst compelled her to move, and she accordingly went to Spring Gardens. Her earnings, however, supplemented by lectures delivered for the Board Schools of Bradford and Shipley, proved inadequate to meet her expenditure, and she has been obliged to file a petition in bankruptcy. The amount is comparatively small, and her repeated efforts suggest that she is a woman of capacity and energy. Nurses who think of risking their small capital in similar ventures would be wise to obtain reliable advice as to locality, expenses of working, and other details before doing so.

### NURSING IN ROME.

"An Old Resident of Rome" writes: "Having read your exceedingly just criticism on the perfectly true report of the excellent work done by the Blue Nuns, or, to speak more correctly, the 'Little Company of Mary,' I venture to add my mite to the information you are accumulating about our nursing arrangements in Rome. I think I may say I know more or less well all the English doctors and nurses, as I am an old resident and much interested in nursing work, and, therefore, I regret to see in your excellent paper no notice of Miss Watson, who opened a small home here the year the American home was closed. She is herself a Queen's Jubilee nurse, a certificated midwife, certificated masseuse, and never takes a nurse with less than three years' hospital training. She is fortunate in having an irreproachable position and a large circle of friends. Although I myself admire immensely the Blue Nuns, and know that every patient nursed by them in the house or outside is always satisfied, yet we have here people to whom the name and dress of a nun is not agreeable, and who think that the training and dress of a lay nurse is preferable. To meet this need Miss Watson started her home, and she has had regular work for six or seven nurses every season. They take their own fees and pay her for their expenses; they live with her and use her name and telephone, and in return she provides them with cases. She has found two or three beds for patients inside quite sufficient, as very few invalids call in a doctor in time to be moved."

### MELBOURNE NOTES.

Mrs Catherine Bishop, the first matron of the Melbourne Hospital for Sick Children, has retired after holding the office for about twenty-five years. During her early years in the position Mrs. Bishop, who had not been previously trained, attended lectures with her nurses, qualified, and took the diploma given by the hospital. Her successor is Miss Hilda Player, for some time Sister in the Sydney Hospital for Sick Children. Miss Player, who was trained in the Prince Alfred Hospital, Sydney, took up duty on March 27th.

### SAD STORY OF A NURSE.

At an inquest held at Troon last week upon Louisa Lyons, a sad story came to light. The deceased was, according to all accounts, a conscientious, cheerful woman of forty-three, and was attached to the Treslothan Nursing Association. No one seemed to have seen her the worse for drink, but evidence was forthcoming to the effect that she frequently consumed large quantities of alcohol. On the night before her death she complained of feeling ill whilst visiting a friend and former patient, and asked if she might stay the night. It was arranged that she should sleep with her hostess, and she retired at once to bed. During the evening she sent for ale, stout, and brandy, all of which she drank, and also some drugs which she procured from the chemists. When her hostess went to bed she found her guest in a heavy sleep from which she could not be aroused. Towards morning, however, after much sickness, she got up and remained sitting holding the leg of the bed, in which position she was found dead. At the inquest the doctor stated that he had discovered traces of chlorodyne in the stomach, and the jury returned a verdict that the deceased had poisoned herself whilst under the influence of intoxicating liquor.

### DEVON AND CORNWALL HOMŒOPATHIC HOSPITAL.

An important feature of the work of this institution is the visiting of the sick poor in their own homes by nurses. Last year, we learn from the annual report, nearly 2,000 visits were paid. Unfortunately, although the patients extremely appreciate the ministrations of the nurses, the support given by the public is limited, and the excess of expenditure over income on the part of the hospital was £253. But for a special effort in the form of a sale of work the deficit would have been much heavier.

### SHORT ITEMS.

At the opening of the rebuilt All Saints' Home for Incurable Boys in Margaret Street by the Duchess of Albany last week, Mr. Higgins stated that since the foundation, sixteen years ago, 34 cases had been entirely cured by medical and surgical skill and nursing. The staff now consists of the sister in charge, one trained nurse, and four young girls, the latter, in return for their services, being trained for domestic service and otherwise provided for.—On Saturday there was a street collection in the locality on behalf of the Bolingbroke Pay Hospital, Wandsworth Common (which is free to accidents), the collectors, for the most part, being small girls under twelve years of age, who were dressed in the costume of a trained nurse.—Dr. Alfred Hill, Medical Officer of Health for Birmingham, writes that the correct designation of the ladies described as "Birmingham Women Health Inspectors" is "Health Visitors." "It is," Dr. Hill adds "in the character of friends and advisers of the people that we wish them to be regarded, and not as inspectors."—The Guardians of Whittlesea have been asked by the local nursing association to subscribe and pay for dressings for the patients nursed by the association who are in receipt of outdoor relief—a very reasonable proposal.—The Lochwinnoch Parish Nursing Association in affiliation with the Queen Victoria's Jubilee Nurses has just issued its sixth annual report. During the year commencing March 9th, 1898, and ending March 8th, 1899, 4,604 visits were paid to patients, 274 patients were attended by Nurse Sawyer and Nurse Collins; of these 214 recovered, 3 went to hospital, 23 died, 4 left the parish, and 30 remained on books. Money donations amounted to £102 12s. 9d., annual subscriptions to £71 11s. 4d., expenditure to £160 7s. 7d.; balance in bank and secretary's hands, £72 14s. 1½d.; deposit receipts, £140.

### District-Visiting Nursing in Obstetric Practice.

By A. Worcester, M.D., Waltham, Mass.

We publish the following paper upon one aspect of the nursing question in America, read before the Obstetrical Society of Boston, as we think it may interest our readers:—

Thirty years ago Dr. H. R. Storer eloquently pleaded for the introduction of trained nurses. There were then in this country no training schools, neither was there any generally recognised need of trained nurses. And even nowadays in midwifery practice the necessity of first-rate nursing service is not sufficiently recognised, or, if so, the efforts made to supply the need are wofully ineffective.

True, the rich have trained nurses in their fashionably infrequent confinements, perhaps in waiting for a month beforehand and in service as nursemaids for three months afterwards. And many moderately well-to-do families half bankrupt themselves to pay to the trained nurse more per week than the breadwinner of the family earns. But for the poor and the average wage-earning classes there is yet almost no provision of proper nursing service: probably not one woman in fifty in this State has a trained nurse in her confinement.

The supply of trained obstetric nurses is still pathetically small. And so long as most training schools persist in ignoring this necessary branch of a nurse's training it is not unlikely that the nurses' registries will continue overloaded with unemployed nurses and that the greatest need of trained nursing will continue unsupplied.

If most of the medical schools likewise ignored midwifery very probably graduates in medicine would have less to do and most certainly women in labour would manage to get on without them. Even in this age of specialists a general education in all the different branches of medicine is deemed essential, and some experience in general practice is deemed advantageous to the physician or surgeon who becomes a specialist. But for nurses a strictly limited, and consequently incomplete, education is thought sufficient and perhaps even an advantage. Not only do the general hospitals graduate nurses who, for instance, have never seen a case of childbirth, but the specialist hospitals also graduate nurses who, perhaps, have never seen a sick adult, or a male patient, or a case of any of the common contagious or infectious diseases. From the hospital point of view this is all well enough. Specialist nurses are what each hospital wants. But nurses who are specialists, not from preference but from ignorance of every other branch of nursing, are not the kind wanted in general family practice. In the large cities, where families are learning to employ specialists in medicine and surgery, calling first this doctor and then that, according to the anatomical location or supposed pathology of their ills, and where scores of nurses, card-catalogued according to their specialities, are patiently waiting for work, the grotesqueness of the present conditions is not fully appreciated. But in the smaller towns and in the country, where specialists do not thrive, the old-fashioned untrained nurses are in little danger of being supplanted by graduate nurses who are either unable or unwilling to take cases as they come. I believe the same is also true as regards the large bulk of the population of cities. In general family practice trained nursing is not likely to be common until nurses are properly trained for such service, and this desirable result will not be attained until training schools are established and managed with the primary purpose of educating nurses for the general practice of their profession. In vew of the recent introduction of trained nursing in this utilitarian country, it is not strange that educational ideals have been made secondary to the needs of the hospitals where training schools have been established, and that hospital nursing has consequently advanced to its present overweening importance. But it is remarkable that trained nurses themselves should continue so ignorant of the history of their own profession and so oblivious of other and equally important departments of nursing as to rank themselves according to the number of beds in the hospitals where they were trained. To such length has this folly gone that the Association of Training School Superintendents admits to membership only such as have been trained in hospitals of over fifty beds, unmindful of the fact that by such a test many of the greatest nurses living—even Florence Nightingale and Elise Aberdieck—are ineligible. With such false standards flaunted before them what hope is there of abatement of the insufferable hospitalism that now hinders many graduate nurses in making their way in private nursing!

In Germany, where the modern profession of nursing began, the great training schools still control and work the hospitals in which the student nurses receive that part of their training. In Great Britain the matron of the hospital is the real, as well as the nominal, head of the whole institution. Only great teachers of nurses are given these matronships, and in the training schools under their leadership the profession of nursing steadily advances. In this country most training schools are mere adjuncts of the hospitals, designed and managed solely for them; indeed, some of the smaller hospitals are in part supported by the student-nurses' earnings in private service. The cart is before the horse, and if not going backwards at least no general advance is being made in our system of training nurses.

Among the graduate nurses, however, there are many encouraging signs of awakening professional spirit, which will most surely lead to higher educational ideals. The medals and diplomas of their own particular schools mean far less to the graduates of to-day than to those of a few years ago. Post-graduate courses for nurses in the specialist hospitals, as fast as introduced, are flooded with applications. Clinics and lectures for graduate nurses are multiplying and improving.

In Canada there has been established, in commemoration of Queen Victoria's Diamond Jubilee, an order of district-visiting nursing that promises to be of immense advantage to the profession of nursing. In this Victorian Order nurses who have been graduated from approved training schools will be trained in the art of district-visiting nursing, which is as different and distinct from hospital nursing as general family practice of medicine is different from practice in the wards and ampitheatre. The underlying principles in both are, of course, the same, but the methods and means employed bear slight resemblance to each other. For this very reason extra training in district-visiting nursing, as now offered in Canada, will bring into due prominence the essentials of the art. After such a course nurses will be less bound to fads and more willing to believe there may be several other equally good ways of working besides their own.

Not least among the advantages of the Victorian Order will be the establishment of an educational standard for training schools. Graduates from only such schools as are approved will be accepted as probationers of the Order. And schools that fall below will thus be forced to rise to the tandard required. But the chief advantage of the Victorian Order is the rescue of district-visiting nursing from its present obscurity.

*(To be continued.)*

### The Nursing of Phthisis in Sanatoria under the "Open-Air" System.

By the Matron of the National Sanatorium for Consumption, Bournemouth.

I.

Although much has lately been written and said about tuberculosis, its causes, and the various modes of treatment in vogue from the standpoints of both medical men and patients, I have not yet seen anything from a nurse's pen describing the "open-air" treatment as it affects her work. It has occurred to me that a short account of the methods adopted, and now in practice, at one of the largest sanatoria in the United Kingdom cannot fail to be of interest to many of the readers of this journal, and may perhaps be of service to nurses who are thinking of working in an institution of this description. In the first place we must remember that we are speaking of sanatoria and not of hospitals. The "instructions to candidates for admission" as patients to this sanatorium (and it is a type of many such institutions) specify that they must be suffering from "incipient forms of disease," and further that they will be admitted for a term of twelve weeks or upwards, the tendency being lately to lengthen this period to four and six months. In consequence, few of the patients are confined to bed, and the great majority of them belonging to the working and servant classes find themselves for the first time in their lives possessed of leisure, and to a certain extent thrown upon their own resources. The nursing, therefore, in many respects, partakes largely of the character of that in a convalescent home. Besides her nursing qualifications, a nurse in a sanatorium must be possessed of unfailing tact and good humour and the indispensable art of "managing" her patients without appearing to do so.

Some structural alterations which have been taking place in this sanatorium recently speak far more eloquently than any words of mine could do as to the immense change that has taken place in this country touching the treatment of phthisis. Not so many years ago a stranger on being shown over the building could not fail to observe the precautions taken to exclude not only all draughts but the very fresh air itself. Double windows were to be found in all exposed positions, outer frames being of the French pattern, only one half of which was made to open, while the inner were of the usual sash description. The other windows in the institution—of the ordinary type—had both upper and lower sashes "blocked," so that by no possibility could they be opened more than a few carefully-regulated inches. Heavy woollen curtains hung not only on either side of each window, but also behind many of the ward doors. These curtains were shaken at the annual cleaning of the establishment, but it was never considered necessary to disinfect them, many of them having occupied the same position for ten and even more years!

But times have changed. Double windows are a thing of the past; "blocks" have been removed or altered; curtains have disappeared; and from the outside the whole building with its widely-open windows appears gaping and yawning as it lies bathed in sunshine. Visitors on being shown round exclaim as they pass from corridor to day-room, from day-room to night-ward, "How delightfully airy and yet how warm!" and this remark brings me to a point of much importance to nurses, viz., the management of temperature in the day and night wards. The nurse must remember that her patients will practically live out of doors, and therefore the temperature of the rooms in which they sleep and have their meals must not be high or the danger of "catching colds" will be materially increased. The day wards, corridors, and dining-rooms should be kept at a temperature of 55 deg. to 57 deg. Fahr., and the night wards should not be allowed to fall below 50 deg. Fahr. A thermometer must hang in a suitable position in every room, and be carefully watched by the nurse. In this sanatorium a book is taken round each night ward at seven p.m. and the temperature recorded; it is then handed to the matron or sister in charge who marks the rooms where fires are to be lighted. As a general rule, with patients who are able to be out of doors all day, we find that a fire lighted at half-past seven and made up again about an hour later heats the room sufficiently well. To guard against the overheating of the rooms by the patients themselves we allow no spare coal to be kept in the night wards. The hot-water system is only used here to a limited extent in the corridors and chapel, for open fires not only heat the wards but aid greatly in their ventilation.

The windows must on no account be closed if the temperature of the room has fallen below the defined limit; it must be raised by building up the fires, and not, as in the old days, by closing tightly the windows. Whilst speaking of ventilation and temperature of the wards I would explain that, except in great storms of rain, the windows in the day-rooms and corridors are never closed, but open continuously as widely as possible. In the night wards, owing to the proximity of the beds to the windows, we are obliged to allow the lower sash to be closed whilst the patients are dressing and undressing, and, during the night, while they are in bed. The upper sash, drawn down to its fullest extent, is never altered except by the physician's directions. Special instructions as to the regulation of windows for patients in bed in the daytime are in all cases given by the medical man, and it is the nurse's duty to see that these instructions are carefully carried out. By the side of every window in the building there hangs a printed card forbidding patients under any circumstances to interfere with the windows.

Amongst the patients admitted to a consumption sanatorium a large proportion will always be found to be suffering in addition from bronchitis and asthma. For these a separate ward must be kept where the "open air" treatment is carried out to a more limited extent. Here we invariably reserve a ward of four beds for such patients, and another for those of low vitality and enfeebled circulation who have recently come under treatment. Of course, in all cases one has to take largely into consideration the home habits of each individual; but in wards of three or more beds there will always be one more sheltered than the others, and the clever nurse will reserve such for newcomers who may thus be gradually accustomed to the treatment, and so advanced by easy stages to the more exposed positions under the very windows. A sanatorium will, of course, be built in such a manner that both day and night wards have a southerly or south-westerly aspect. The furniture must be simple and easily moveable to permit of the cleaning of both it and the room floors; and if the nurse bears in mind that the inhalation of dust containing the tubercle bacilli or its spores is the common cause of the disease in adults, she will see how very necessary it is to avoid "upholstered" furniture, which affords innumerable lurking places for the enemy. Thus, in the night wards wooden or cane chairs and wire mattresses will be the order of the day; if the floors are polished, Ronuk or some other sanitary polish may be used, but if they consist of plain boards they should be thoroughly scrubbed once a week with an antiseptic soap. Strips of Dutch matting, 45 in. by 27 in., which can easily be shaken, serve instead of carpet by the side of each bed, and if of a bright colour, will add materially to the cheerful appearance of the room. Marble-topped washstands are luxuries out of the reach of sanatorium patients, but a strong wooden one painted over with a couple of coats of enamel forms a very good substitute, having a remarkably neat appearance, standing much scrubbing and being easily renewed when shabby with another coat of paint. In the day-rooms the furniture at first presents a difficulty. Phthisical patients are generally thin and do not appreciate hard seats and couches, but we have the chairs and sofas covered with a smooth soft leather, avoiding the slippery variety, and over that again washable covers of a cheerful chintz. Above all things, upholstery of the "buttoned down" description must be avoided; it is a veritable germ trap. Woollen curtains and tablecloths must be tabooed, and the floors of the day-rooms either polished, with moveable mats placed here and there, or covered with cork linoleum.

Some authorities recommend the walls of the various wards to be distempered. I think that this is a great mistake, for even the best distemper tends to peel off, and the result is an uneven surface affording innumerable hiding places for germs. Besides, any nurse who has assisted at a "ward wall sweeping" is not likely to forget how much dust collects on the surface of any wall, and this dust, even if it be not visible, must be frequently removed, a manifest impossibility if the walls be distempered, for then they can be neither washed nor swept without ruining their surface. The difficulty is surmounted if the walls be painted, a more costly process, but infinitely cleaner, as they can be washed as often as desired without injury.

*(To be continued.)*

### The Matron's Corner.

### WISE AND JUST RULE.

It has been said, and, I fear, with some justice, that a woman does not make a good ruler; that she is too apt to become a tyrant in a position of authority. It does sometimes seem as though a woman could not bear the weight of government. She is apt either to become a tyrannical autocrat, or she holds the reins of rule so loosely that they fall into others' hands.

To be a wise and just ruler is not easy; it involves no small amount of self-sacrifice. It implies loneliness. A matron who is a good ruler cannot make intimate friends with any of her staff. Each individual must be treated with equal impartiality, for any other line of treatment would lead to jealousy and bitterness. Again, a matron must set aside her pet prejudices, her private likes and dislikes. She cannot indulge in her antipathy towards this person, her sympathy towards that. From the nature of her office she must cultivate a wide toleration with all sorts and conditions of mind and character, a kindly understanding of temperament opposed to her own.

We are inclined, as we grow older, to become more suspicious of our fellow-beings. Might not all rulers adopt the custom of that wise headmaster, Dr. Arnold, of Rugby, who made a rule of always trusting a boy until he was actually discovered to be unworthy of trust?

I have observed how frequently hospital authorities adopt a suspicious attitude towards their subordinates, and it seems such a pity. Nothing makes people more liable to deceive and to act in opposition to rule than the consciousness that they are being watched with distrustful eyes.

Trust those under you; appeal to their sense of honour. Never mind if your trust is over and over again betrayed. To trust is a million times better for your own character and for the character of those you rule than to suspect. It cannot be either wise or right to turn a hospital into a miniature Russia, with a system of espionage, an atmosphere of suspicion.

If a matron is herself the soul of honour, it is wonderful how soon dishonourable acts pall upon her staff. To be absolutely just means that your heart must never govern your head; that you must invariably make yourself see two sides of every question; that you must hold the scales of justice without hasty balancing.

Because your word is law it behoves you all the more to cultivate scrupulously what we women are said to lack, the spirit of equity.

A true "leader" is a rock of firmness—strong, honourable, upright, single-minded, and unswervingly just.

### Typical Patients.

### THE PATIENT THE GODS LOVE.

To begin with, she had all the "tuberculous virtues." She was only five, and a small person at that, but when her mother brought her into hospital, and wept much at parting from her baby, the mite gravely told her not to cry, that visiting day would soon come round, and that she, No. 10, intended to get well and fat very quickly, by dint of taking all that was given her, "not like brother Billy, who wouldn't eat his victuals, and dieded." Then she lay quietly down, hugging a time-worn doll that nurse put into her arms as consolation, looked contemplatively round the ward, and finally asked sister, who was busy near her, if she "ever threw stones at little birds?" Being reassured on that point, she proceeded to relate the family history to a motherly body in the next bed, giving many particulars of the defunct Billy and the two brothers at home. "Mother hasn't many children," she explained; "they are so much trouble." She was very quiet after tea, and night nurse, when she came on duty and went round the dimly lighted ward, heard a plaintive wail, "Oh, kiss me, please, and kiss my dolly too!"

Soon she became the pet of the ward, and a privileged person. She was a pretty little soul, with delicate features and a waxen complexion, her golden hair was her special pride, and she treasured above everything a piece of pink ribbon with which her great friend, the night nurse, tied up her curls in the morning. The doctors were always greeted with her sweetest smile; she would help nurse take off her nightgown, and sit perfectly still while the examination was going on; but sometimes there was a wistfulness in the look she fixed on the surgeon's face that made sister wonder if to that childish mind had already come the fear of following brother Billy.

Theology had much charm for her. She had evidently been brought up in a Calvinistic atmosphere, for her predictions as to the ultimate destination of patients who would not eat their dinner were enough to strike terror to the hearts of such offenders. Her sympathies were catholic, however, for she struck up quite an intimacy with an Anglican "brother" who visited a patient in the same ward. His girdle she admired immensely, and when sister gave her the cord of an old dressing-gown to tie round her own waist she was overcome with delight. Soon after No. 20 died the little maid was heard to wonder "how she liked Heaven," and to suggest in the next breath that No. 19 was "nearly old enough to go, too."

As the weeks passed by the wee white face grew smaller and whiter, and after the second operation her shrill little voice was seldom heard. Sometimes she cried softly, with half-smothered, tired sobs, pitiful to hear, but when nurse would anxiously inquire what was the matter, she always replied, "Nuffin," with a big effort to look unconcerned. "Dolly," in the last stage of decay, and minus an eye, was still her comfort, and was regarded by her as the panacea for all the ills the ward was subject to, and sister would be gravely requested to carry Dolly as a temporary loan to any patient whose woes aroused her sympathy. Generally, she lay all night—more often awake than asleep—in the same position, one tiny, transparent hand under her cheek, the other resting maternally on Dolly's tousled head. And so she lay one night when nurse made her first round, but she did not want even night nurse any more; her little face was peaceful now, the weary lines traced by pain between her brows had been gently smoothed away, and the pale lips seemed almost to smile, for on them had been laid "the kiss of God."

### Across the Seas.

### NURSING OUTSIDE JOHANNESBURG.

I HAD only just started on my first lesson in massage from the night super at the Johannesburg Hospital, when I was honoured by a visit from the matron, accompanied by the doctor from the G.M. Co.'s Hospital. Could I go out to the hospital? The housekeeper was ill, and the nurse-matron, with the help of two black " boys,' was trying to do the housekeeping (no sinecure in those parts) and the nursing of seven or eight patients, mainly typhoid. I was only too delighted to meet their wishes, as I was anxious to see something of mine life, and within a few hours I had taken train for B——, a little place about ten or twelve miles from Johannesburg. From there I took a Cape cart, which landed me after a drive of an hour or so at a two-storied double house, with a verandah running along the front. There was an effort to attain to a garden. A barbed wire fence ran round a good-sized piece of veldt, where were three or four gravelled paths bordered with flowers; the rest was mainly unreclaimed veldt, except here and there a bed or a patch of vegetables.

I was greeted at the door of the first house by Nurse W., who showed me my little bed-room, opening out of one of the wards and just big enough to hold bed, chair, dressing-table, washstand, and box. Never was I so glad before that I had learnt to travel with little luggage. They had been waiting supper for me, and after my drive across the "veldt" I was delighted to get it. Then I went in search of nurse, who took me to see the three little wards, but assured me that I should have nothing to do till next day, and had better secure a good night's rest. When, however, I heard that the patients were to be left for the night in charge of a black boy, who would call Nurse W——— if necessary, I did not feel that sleep would come easily to my eyes. I did not then know "Jim," the hospital boy, as I got to know him later.

At twelve o'clock there seemed to be a thundering knock on the door leading from the ward, and jumping out of bed I slipped into my dressing-gown and went to see what could be the matter. All appeared quiet. The black boy was lying on a mattress by the bedside of one of the patients, only the man in the bed across my door seemed restless. I went over to him, found he was asleep, but that every time he turned over he banged his knee against my door, which, without waking him, sounded to me like thunder. I roused again later to hear my bed-room door closing stealthily behind someone who was just leaving the room. This time I did not disturb myself as I heard Nurse W—— about and knew that all must be well. Next morning I found that Jim had come into my room by mistake to call her, thinking that she still slept there.

After half-past eight breakfast I was initiated into my work. The patients were then all typhoids, two very seriously ill, not expected to recover, two convalescent, one of them the man who had roused me from my sleep the night before, the others in bed, but doing fairly well.

Before breakfast the wards had been swept and tidied by the hospital boy. Jim had worked in the large hospital in Johannesburg, was a really good nurse, and very trustworthy. He could read and write his own language and talk English fairly well, without making use of any superfluous words. There were then the patients to wash, the beds to make, the wards to dust, and the flowers to arrange, for we had a very perfect and smart little hospital.

The surgery was nicely fitted with glass cupboards for the instruments, a good operating table, and satisfactory arrangements for sterlising the dressings, which was invariably done as carefully as in any large London hospital. Our floors were scrubbed several times a week and washed over every day, and our walls were all painted up to the ceiling.

I was able to get a little rest before dinner, to which meal an ex-German officer (holding some official position in the mine) came daily. Later, Prince ——, a well-known shot in Johannesburg, a Russian, not unknown in European society, turned up, with a French friend, for a cup of Russian tea, which Dr. —— rightly prided himself on making to perfection. The prince entertained us with stories of adventure and travel in many lands. Then to the wards again, and a little rest in my room, necessary in the afternoon in a hot climate if you intend to keep in health. Later, tea on the verandah, with visitors again, this time one Englishman to leaven the foreigners. Afterwards there were the patients to be made comfortable for the night, and by the time that was finished supper was ready. Then a walk round the premises, leaving Jim in charge, and telling him to send Tom, the house boy, for us if necessary. To bed about ten o'clock. Tom was left in charge of the hospital, his mattress being by the side of the patient who was most seriously ill, with instructions to call me after twelve o'clock, or before if necessary, to administer the stimulants: No native can be trusted with brandy. Next day there was a bad accident in the mine. The rain had loosened some rocks, pieces of which had fallen on, and severely injured, several natives. The doctor, Nurse W——, and Jim went off to the native hospital in the compound, and left me for several hours to my own devices.

The native hospital attached to the compound where the natives live is a large room, about forty feet long by twenty broad. The beds are merely stretchers, the natives bringing their own blankets; pillows and sheets are not used. Surgical cases were capitally treated by the doctor, and under native care—the dressings, of course, being done by the doctor—got on very well indeed. The medical cases were, I fear, less successful. It was impossible to spare time to instruct the natives in the art of nursing typhoids or pneumonia. These, with mercurial poisoning, formed the bulk of the cases in the native hospital. All that was or could be done was to give a slight change of diet, viz., from mealy meal, or Indian corn porridge, to boiled rice. It is said that some survived the treatment, but I never inquired too closely into the matter. Our white patients were fed only on tinned milk; cow's milk (as fresh milk is called at Johannesburg) was not to be had in so large a quantity as we required. I stayed on at the hospital for two months nursing, and our sick folk did well. We had enough work, though not too much, plenty of society, and no worry. Somehow, things seem to go more quietly abroad than in England; no one troubles unless there is real need, and life proceeds quietly and peacefully from hour to hour and day to day, under continuous sunshine and blue skies; consequently, smiles are much more prevalent than in England.

### A Correction.

WITH reference to an article in a nursing contemporary correcting certain statements made in these columns respecting the International Congress of Women, in the course of which Miss Maule is mentioned as "the editor of the 'Nursing Mirror,'" this is an entire mistake. Miss Maule is not the editor of the "Nursing Mirror," neither is she in any way responsible for the statements made concerning the nursing section of the Congress in this paper.

### To Nurses.

IN order to increase and vary the interest in the *Mirror*, we invite contributions from any of our readers in the form of either an article, a paragraph, or information, and will pay a minimum of 5s. for each contribuion. All payments are made at the beginning of each quarter, *i.e.*, January 1st, April 1st, July 1st, and October 1st.

### Echoes from the Outside World.

AN OPEN LETTER TO A HOSPITAL NURSE.

I expect an increased feeling of loyalty has found its way into the hospitals this week, as it has all over the country. As a general rule, the Queen's Birthday, except to those who are hoping to get a title, is of very little importance in England, though I know from friends in the Colonies that there they look upon it as a very special event, giving the school children a holiday, and organising treats and entertainments galore. But this year May 24th has been quite a day of enthusiasm in England too. Birthday services have been universal; all the London Board School children have had a half-holiday; the children of a larger growth employed in some of the large City warehouses have been "let off" a few hours earlier; the Phœnix Park murderers have been released; and all convicts enjoyed a half-day holiday. Talking of birthday honours, if you are an admirer of Rudyard Kipling, it may interest you to know that it is very probable after June 3rd—when the Birthday *Gazette* will be published—you may have to speak of the author of "The Light That Failed" as "Sir Rudyard." Everyone, I believe, will be glad if the rumour turns out correct, for Mr. Kipling's recent illness must have proved to all how many friends his books have gained for him—far more than he or the outside world had dreamt of. Another name we should be pleased to see with a prefix of distinction is that of Henniker Heaton. Nurses' pence are too hardly come by for them not to be grateful to the man who has enabled them to communicate with their distant friends at the same cost as those in Great Britain, for the modest penny.

---

All our preconceived ideas with regard to the Muscovite Empire seem going to the winds. As far back as our childhood's days the very name of Russia has been associated in our minds with the fear of war, and the horrors of exile to Siberia. The Peace Conference, which is being held at the Hague, is entirely the outcome of the proposal of the Czar to abolish war, and if ever that blessed time should come when the nations cease to do wholesale murder, it will be to the Autocrat of all the Russias that the boon, under God, will largely be due. Now His Majesty has called a Commission, under the presidency of the Minister of Justice, to consider whether some other punishment could not be substituted instead of transportation to Siberia, with its attendant terrors of work in the mines, and hard labour under insanitary conditions. Since the seventeenth century the system of transporting criminals has been carried on, but the time has at length come when Siberia itself—and, as many of us think, the cause of Humanity—demands that some alteration should be made. In a few years' time it seems probable that, instead of the name of Siberia striking despair into the hearts of the Russians, it will represent to them nothing worse than a colony where civilisation is advancing apace, and where there is room for those who may have failed at home to start a fresh life with new hope.

---

Last week I alluded to the young actress who, though related to an Earl and having had provincial experience, was willing—and glad—to be a walking lady at a London theatre. But there are ladies of higher degree who find it necessary, in order to ensure success in their enterprise, to do a little of the drudgery. You have probably heard of the Countess of Warwick's School of Needlework. The product of her workers is sold in New Bond Street, and Lady Warwick has learnt that, though the employés in whose hands the business has hitherto been left may have done their best, it is very desirable that she should sometimes attend at the shop herself. Accordingly she has arranged to be there the first and third Tuesdays throughout the remainder of the season, and on Tuesday she served behind the counter for some time. The result was that the shop was crowded for the remainder of the day.

---

It is a far cry from an oratorio to a carriage-shaft, and yet it is in the character of the inventor of a life-saving apppa-ratus to be attached to carriages that Sir Arthur Sullivan is to appear next week. You may remember that the late Countess of Lathom, who was a most intimate friend of the well-known composer, was killed in a very sad manner, owing to her horses taking fright. Ever since then Sir Arthur has been trying to invent some means of averting the fatal results which frequently follow a sudden fright on the part of the animals. The "Sullivan Safety Shaft" will enable the horses to be released from the carriage at a moment's notice when necessary, and should it work satisfactorily it would do a good deal to allay the nervousness of those who, though they may perhaps never be called on to experience a carriage accident, suffer from a continual haunting fear which spoils the pleasure of every drive.

---

The extreme partiality of the female sex for the cup which cheers, but does not inebriate, has for years been a joke which the lords of creation have harped upon with much glee. And now a case has occurred in which the medical evidence proved that the nervous system had been nearly destroyed by "tea poisoning" owing to the enormous amount of the beverage consumed, and the coroner accordingly has given a verdict of "Killed by excessive tea-drinking." Yet the victim, after all, was not a woman, but a man! He was a furniture dealer, and succeeded in destroying himself at thirty-eight years of age, but this is not a matter for wonder, considering that he often took eight cups of tea before leaving home in the morning. I do not know any woman who goes to such a fearful extreme, but I do know dozens of women who injure their health by drinking too much tea. A cup of tea before rising (often without anything to eat), two more cups at breakfast, a little "brew" at eleven when at all tired or out of sorts, another at midday lunch, followed by two more cups at afternoon tea, and a final after dinner at night, is the allowance of more than one teetotal friend. And then the poor dears pat themselves approvingly on the back when they hear of a dipsomaniac, and congratulate themselves that they at least do not "drink to excess."

---

Most sensible women have to acknowledge that the children of this generation are brought up too luxuriously, and are allowed far too many sweets and too late hours to be good for them. But the gentleman of threescore years who took the occasion of the wet Bank Holiday to write to one of the dailies to say how he managed to keep always in health, working from eight a.m. to ten p.m. at a sedentary occupation, with only a walk on Sunday, and no yearly holiday, was surely a little too severe on the small mites. He would "throw aside sugar-basin, jam-pot, sweets, confectionery, pastry, and intoxicants, none of which children should ever be taught to use." By all means let the three last items be struck off the nursery mènu, but *no* jam, *no* sugar, *no* sweets makes my heart ache for the juvenile joys which would vanish. As I heard a little girl say the other day, "Not much dam, mudder, but just an 'ickle to make the bwed yoo nikk'd."

### A Book and its Story.

### THE LATE PROFESSOR PALGRAVE.*

The name of Francis Turner Palgrave, late Professor of Poetry at Oxford, is a distinguished one in the world of letters, and a household word to those who know it, perhaps, only through his "Golden Treasury" and other valuable collections of verse. Poet, philosophor, critic, an accomplished linguist and acute observer, he stands out before the public in "His Journals and Memoirs," recently edited by his daughter, not only as a man of rare mental gifts, but also as one of high personal character, whose absolute "selflessness" and attractive loveableness, united with a strong manliness, endeared him to all with whom he came in contact. The eldest son of Sir Francis Palgrave, K.H., he was born at Yarmouth in 1824, and like many notable men he was of Jewish descent on his father's side. The name of Palgrave was assumed by his father on his marriage in 1823, being the name borne by his mother-in-law. Sir Francis himself was a man of unusual ability, and, after a life marked in its earlier years by severe reverses in consequence of the failure of his father's fortune, he became distinguished as a historian and antiquarian. In 1832 he was knighted, and a few years later found him appointed Deputy Keeper of Her Majesty's Records, which appointment he held until his death.

Professor Palgrave was singularly fortunate in all his domestic relations. His childhood was blessed by the loving care of a cultivated, charming mother, whose devotion to her children is seen in her letters and the deep reverence and affection by which she was regarded by her family. Like his friends Gladstone and Tennyson, he was happy in the possession of an adored and devoted wife. Throughout his life, when moved deeply by joy or sorrow, he sought expression in verse as "a relief, which is the natural outcome of a poetic nature." These are the closing stanzas of a poem written during his short engagement to Miss Grenville Milnes, whom he married in December, 1862:—

"Her eyes spoke peace; and voice and step
The message of her eyes repeated;
Truth halo-bright about her brows,
And faith on her fair forehead seated;
And lips where candour holds his throne,
And sense and sweetness are at one;
I look and look; and something there
Is fairer than the fairest fair."

"No more beautiful description of my mother," adds Miss Palgrave, "could be given. A letter from Lady Carlisle, written twenty-eight years afterwards, proves that time endorsed the lover's verdict, when the dearly-loved wife and mother entered into rest: "I am deeply troubled at your great loss. . . . I am so sad for you; so very, very sad. There never was such a marriage—such tender love, such perfect companionship, all sundered and lost. And the children left without her great warm beautiful love! Oh! the sorrow when a union like this is shattered! . . . . Yet blessed above most marriages yours has been, a true heavenly marriage, and it has made all life holy to you. If it is over at least it has been perfect."

Surely no one has more beautifully expressed their sorrow at the loss of one beloved than did Francis Palgrave, when a year later he writes:

"O Love, whose very thought towards me was love,
Thine heart in mine beating through joy and woe,
Star sent from heaven to still life's storm and stress,
Steering the boat now rudderless;
How should'st thou quit me so,
From thy dear presence parted
Broken-hearted.

O child of God, my counsellor, strength, and stay,
Yet very woman in gay gentleness;
Wife, mother, child, at once I saw thee move,
Sweet alternation of one love;
With gracious, grave caress,
Holding me by thy spell
Irresistible.
Loved, honour'd past all words, thy prayers I pray,
My saint, my own!"

We have spoken of the happy childhood of Professor Palgrave, and not only had he and his three brothers the blessing of affectionate, judicious parents, but they had also the privilege of being reared in a God-fearing household.

"Both parents were eminently pious people, and were accustomed constantly to bring forward matters of religion to bear upon the occupations and amusements of their children. 'To point a moral' was one of the first principles in the father's training of his boys. Always imbued with strictly Church principles, they were much influenced by the Tractarian movement, while it is easy to trace the effect which the training of J. H. Newman exercised on their lives. . . . The boys were brought up to go to church on weekdays, and on Sundays, besides generally attending two services at Hampstead, they would often walk in the evening to Christ Church, Albany Street. It was Frank's amusement to construe the sermon into blank verse whilst it was being delivered."

"The greater part of his childhood was spent at Hampstead." But he had no love of London, although from necessity the greater part of his life was spent there, varied later by frequent visits to Lyme Regis, where in 1870 he purchased a property known as "Little Park," having historic relations since Charles I.'s days. From an engraving we see that it must have been a charmingly irregular pile of gabled masonry, formerly two cottages, and just the place to please an artistic poetic eye, such as his.

We have dwelt, perhaps, too much on the social aspect of Palgrave's life, and earlier in this review should have mentioned that, educated at Charterhouse, he and his gifted brother Gifford at once attracted the attention of Dr. Elwyn, the head master, when, in 1843, they were attending the school "coming daily from Hampstead." "The wonderful abilities of the two eldest Palgraves and their love for poetry and books" was remarkable. From Charterhouse Francis was elected scholar of Baliol, and his brother soon after became a scholar of Trinity, Oxford. For five years Francis held the post of vice-principal of Kneller Hall under the present Archbishop of Canterbury, Dr. Temple, for whom he entertained the greatest admiration and affection. Besides other appointments, he was private secretary to Lord Granville and Gladstone, held an official appointment in the Education Department, where he was a contemporary of Matthew Arnold, and in 1886 succeeded Professor Shairp as professor of poetry at Oxford. An intimate and beloved friend of Tennyson, Browning, Clough, Matthew Arnold, and many other distinguished men, his life abounds with reminiscences alive with interest. There are interesting references to Mr. Gladstone from time to time, and constant ones to Tennyson, "whom I consider, taken all in all, to be the best talker I have ever known." Carlyle is described as "rough and genial," a man of great sympathies, hence "Weltschmerz," otherwise his opinions, historical and otherwise, were wholly antagonistic to his own. We regret the impossibility of doing justice to this very interesting and well put together memoir, which our readers will find of absorbing interest. Professor Palgrave died in London October, 1897. He never recovered the shock of his wife's death, and was in failing health until the end came.

* "Francis Turner Palgrave: His Journals and Memoirs of His Life." By G. F. Palgrave. (Longmans, London.)

### A Change of Diet.

Of late years the feeding of the sick has received special attention from the medical and nursing world. The importance of carefully-regulated diet, and the share it has in helping the invalid to regain a normal condition of health, are well known to those who are in constant attendance in the sick-room. In the mind of the medical man the diet list shares the honours of the prescription, and his directions with regard to the quantity and quality of the nourishment to be given are obeyed with as much care as those which regulate the medicine prescribed, and the times at which it is to be administered.

The nurse who has gained her experience in a good training school knows at a glance the class of diet her patient will need. While a serious illness runs its course very little is required of her in the way of sick cookery. Milk forms the chief support of the invalid; the old-fashioned gruel is obsolete, and even the standard beef-tea is often displaced by chemical preparations. At the beginning of convalescence, however, there is a change in the doctor's orders. The patient must be fed up that he may regain his strength, and it is necessary that his appetite should be tempted in order that he may relish and digest his food.

In convalescence after fever and some other cases there is little difficulty in getting the patient to eat; the nurse has rather to restrain than encourage his appetite. But in the many cases of illness that are followed by extreme physical weakness, and, moreover, by nervous depression, the case is different. A patient of this class will trifle wearily with the food on his plate and leave it. He declares that he cannot eat—the sight of food makes him feel sick; the boiled chicken is insipid, and he is tired of milk puddings. In his own words, he "wants something he hasn't seen before, something with a taste to it."

It is at this point that the resources of a nurse often appear to fail. Her patient refuses the simple dishes that she has been accustomed to give at this stage of recovery, and her knowledge does not always extend farther. She has been taught that flavours and "made dishes" are harmful, and therefore to be avoided. How is she to humour the appetite of her charge and yet to keep within the narrow limits of orthodox diet?

The right thing appears to be to widen the limits, and to make the food, in the patient's own phrase, "interesting." Sick cookery seems at present in its initial stage, and the instruction given is, of necessity, confined chiefly to the proper preparation of those comparatively few dishes that custom and experience have shown to be suitable to the generality of the cases. It is acknowledged that certain articles of food are more likely to benefit the convalescent than others of a less digestible kind. It is a difference of preparation that must be sought, rather than a difference of material.

After many cases of serious illness milk remains for some time a staple article of diet, but the daily milk pudding becomes monotonous and at last unbearable. An ingenious nurse will discover that there are many other ways of using this valuable food that have greater novelty and are less insipid. Soups in which milk takes the place of stock are nourishing, digestible, and have the merit of being quickly prepared. Vegetable marrow, cauliflower, celery, and other vegetables can be used as a basis. White soup made with Jerusalem artichokes is delicious, and easily assimilated by the invalid. An excellent soup can be made with tomatoes and milk, but it must be passed through a hair sieve as the little seeds of the fruit are apt to irritate a delicate stomach. These soups can be varied and enriched by adding a small quantity of stock made from fresh meat, or in other cases by the addition of the yolk of an egg or a little cream. The American dish known as milk-toast is, in its simpler form, a digestible and savoury way of serving milk.

Examples might be easily multiplied, but this article is intended to be suggestive rather than technical, and its limits do not allow of more than a cursory reference to the recipes. A glance into one of the many manuals of cookery that are issued yearly will show that there are many dainty dishes of chicken, fish, or egg that can by a little alteration be rendered fit for the delicate stomach of the convalescent. That every trained nurse should understand enough of gastronomy to enable her to devise means to tempt the appetite of her patient goes without saying, and the excellent lectures on food and sick-cookery that are given under the auspices of various societies enable those whose knowledge is limited to obtain a practical as well as a theoretical acquaintance with the art.

### Nurses and County Council Lectureships.

One of the latest departures in women's work is the establishment by the various county councils of lectureships in health and hygiene. Nowhere are the lectures more appreciated than in the mining villages of Northumberland and Durham—those long rows of brick houses, all as alike as peas in a pod, with piled-up dustbins and open sewers against which nurse wages wordy warfare.

"Art thou gaun to hear the nurse to-neet? Her's preaching on fevers and sic-like"—the miners say to one another, glad of anything to pass the long, uninteresting evenings.

One night I advocated cremation, and the animated discussion which followed proved an excellent advertisement, for on the following night a large audience thronged into the hall, which had hitherto been but sparsely filled.

Usually some local doctor or popular member of the council accompanies the nurse to the platform, and after somewhat timid reference to "Mr. Chairman, ladies, and gentlemen," the lecture is commenced—generally some simple explanation of hygiene laws and principles—and after the lecture questions are asked and answered.

One of the most popular of our local medical men was most unwilling to take any prominent part in our lectures, although heartily in accord. He was, however, persuaded to take the chair, where he seemed to feel decidedly uncomfortable—the cynosure of all eyes. When he moved a vote of thanks to the lecturer, a great burly pitman shouldered to the front.

"An' I move a vote o' thanks to our doctor, lads," he cried, with a broad Northumbrian burr. "D'ye mind the great explosion and what he done for us. He was the young doctor then, but he is the old doctor now; but we'll never forget 'im." A ringing cheer followed, with a deafening thunder of hobnailed shoes.

On one occasion we had great difficulty in finding a hall for our lectures, and gladly accepted a Primitive Methodist chapel. A bent old man who sat in the front pew appeared to think he had come to hear the perfervid oratory of a woman preacher. Being stone deaf, he did not understand the new gospel of health, and he accompanied the lecture with a running commentary of fervent responses, his ejaculatory "Glories!" and "Hallelujahs!" causing great embarrassment to the lecturer and amusement to the audience.

But the most helpful meetings are the afternoon lectures to women, when crowing babies are much *en évidence*, other women bringing knitting and sewing, while nurse discourses on poultice making, rickets, and croup, and sees the fruit of her labour when some young mother coaxes baby to resign the pie-crust which it has been contentedly munching with its toothless gums.

The work is well paid and interesting, but it is intermittent and needs robust physique and considerable power of endurance, as the constant travelling entails considerable fatigue.

### Examination Questions for Nurses.

Nurse M. E. Davies is the winner of the first prize, and Nurse Despard of the second. The answers this month have been very good, showing thorough acquaintance with the subject; so many being above the average there has been some difficulty in selection. One point on which great importance should be laid and which both these ladies seem to understand, is the extreme care necessary for the nurse to use in disinfecting herself before taking her daily walk. I fear this is a point too often neglected. I should like to point out to Nurse Davies that a solution of 1 in 20 is preferable to 1 in 40 for her protecting sheets. I had a few papers couched in very learned language, but all this medical erudition is a poor substitute for practical knowledge and common sense. It is a curious fact that not one single answer among the 179 I received gave any reason for choosing a room at the top of the house. I should like this matter cleared up. Almost all candidates stated their desire for a top room, but no one said why they wished it. I shall therefore add a supplementary question to that for June.

With the June questions our examinations will stop until October, when we hope to continue as usual. I shall be obliged if candidates will put their name and address on the competing papers, and not on separate slips. These are apt to become separated and lost when so large a number of replies are under consideration.—[The Setter of the Papers.]

First Prize.

On going to nurse a case of infectious fever, if the patient is not isolated when you arrive, and you are allowed to choose the room, a large airy room on the top floor with two windows and a fireplace will be best. Plenty of light, air, and free ventilation are absolutely necessary both for patient and nurse. Remove all carpets, curtains, and as much of the furniture as possible, especially drawers containing articles likely to be needed by the rest of the household. The carpets should also be removed from the landing and adjoining room, which will be needed for many things. A large sheet must be hung up over the door of the sick room, and if it can be managed, one over the head of the staircase to shut off the other part of the house; both these sheets must be kept wet with carbolic solution, 1 in 40. All soiled linen must be put straight into carbolic solution. This should be well and carefully mixed or the crude oil floating about will destroy the linen and burn your hands. After being soaked in the solution some hours the linen should then be well boiled. Blankets and flannels which cannot be boiled should be put into 1 in 20 carbolic solution and afterwards washed with carbolic soap. The rooms should not be swept or dusted in the ordinary way, but be kept quite fresh and clean by being washed daily with some disinfectant, and the furniture wiped with cloths wrung out of the same. The nurse should ask the doctor what he would like used as a disinfectant for washing the patient, and what oil when desquamation commences. The mouth and teeth will need special attention, and some antiseptic mouth wash used frequently. Old pieces of linen should be used instead of handkerchiefs, and then burnt. A little colourless disinfectant should be put into the bed pan and urinal before use; if dark-coloured disinfectants are used you cannot note colour of motions. All excretions should be well mixed with strong disinfectants and allowed to stand a little while before being emptied, and in country places where there are no w.c.'s, but closets often connected with ashpits, chloride of lime should be used freely. A bowl with 1 in 30 carbolic solution should be kept in the room ready to plunge the hands in after attending to the patient. The thermometer should be kept on cotton wool in a vessel with carbolic solution. When desquamation has quite ceased the patient must have two or three carbolic baths, washing the hair and using nail-brush and bath-rubber freely. The final bath must be taken in a room in which fresh clothing is all laid ready, so that the patient may not come in contact with anything used during his illness. All vessels, glasses, and spoons needed for the patient must be marked and kept for his sole use during the illness; all food left over should be at once burnt or otherwise destroyed. The nurse must pay great attention to her own personal cleanliness, and must not go amongst other people without changing her clothing, and not then if it can be avoided. She must pay strict attention to her hands and nails, and use an antiseptic mouth wash before taking her food. In the country she will most likely have to carry out the stoving of the rooms and her own clothing at the conclusion of the case. Before leaving she must take at least three carbolic baths, washing her hair, and after the final bath putting all fresh clothing on. All books, toys, and other articles used by the patient or in the room that cannot be boiled or effectually disinfected should be destroyed, or toys and books if first stoved may be put in a tin box and sent to a fever hospital.

Second Prize.

To ensure the non-spreading of an infectious disease as far as is possible in a private house, the strict observance of two things is essential, viz., complete isolation and thorough cleanliness. Where a choice of rooms is possible select one as far apart from the rest of the house as you can. Remove all unnecessary furniture, carpets, curtains, &c. Hang a sheet rung out in carbolic 1·20, or any disinfectant available, over outside of door, and sprinkle floor and landing with the same from time to time. All soiled linen, except such as can be burnt after use, must be put into a 1·20 carbolic solution and left to soak some hours before being taken away. It should then be removed and washed at once, and, if possible, boiled for some time, but it must be kept apart from everything else. All requisite articles for food, &c., should be kept either in the room or on a table outside the door, and must be washed and kept clean by the nurse. Nothing must be allowed out of the sick-room without first being disinfected as thoroughly as is possible. As regards the nurse, before going out she should wash and disinfect her hands well, and change her dress for one not worn in the sick-room at all. She must not enter any other rooms in the house for fear of carrying infection. On patient's recovery, before leaving his room he must be thoroughly disinfected, a carbolic bath being given, and the hair also well washed. An entirely fresh set of clothing must be worn, and nothing infected used or taken out of the room. Before quitting the case the nurse must see that all infectious articles, such as bedding, clothes, &c., are left ready for fumigation, and where necessary disinfect the room herself. Afterwards she will have to disinfect herself and clothes before mixing again with the outside world.

Question for June.

Give your ideas as to the selection of a bed-room for an invalid likely to be confined to bed for a long period. Also as to the kind of bed best for the purpose (speaking generally), its position in the room, the arrangement of windows, doors, light and ventilation, and also of general furniture.

Supplementary Question.

State your reasons for selecting a room at the top of the house for cases of infectious disease.

### Appointments.

Birmingham and Midland Counties Sanatorium.—Miss Mary Gardner has been appointed Lady Superintendent in the place of her sister, who resigns through ill-health. She was trained in connection with the Mildmay Nursing Association at Crumpsall Infirmary, Manchester, and subsequently held appointments as staff nurse at the Birmingham General Hospital (two years), sister at Cardiff Infirmary (two years), matron of the Llandudno Sanatorium for Women (one year), and deputy matron, Nottingham Isolation Hospital (three years). For the past two years have been matron of the City of Norwich Isolation Hospital.

Basford Sanatorium, Notts.—Miss Clare Hickmott has been appointed to the post of Matron, which was rendered vacant by the resignation of Miss Edith Pringle. She was trained at St. Bartholomew's Hospital, and became assistant-matron of the Metropolitan Convalescent Home at Walton-on-Thames. She left this for the more important post of assistant matron of the Western Fever Hospital, under the Metropolitan Asylums Board, where she subsequently performed the duties of matron during a period of three months.

The Countess of Dufferin's Victoria Hospital, Calcutta.—Sister A. M. Rawlings has been appointed Matron. She was trained at the Edinburgh Royal Infirmary, and afterwards held the appointments of sister at the Marylebone Infirmary, National Hospital for Paralysis and Epilepsy, London, and General Plague Hospitals, Poona and Bombay.

### Everybody's Opinion.

[Correspondence on all subjects is invited, but we cannot in any way be responsible for the opinions expressed by our correspondents. No communication can be entertained if the name and address of the correspondent is not given, as a guarantee of good faith but not necessarily for publication, or unless one side of the paper only is written on.]

### OUR CONVALESCENT FUND.

THE following has been received from Nurse Katherine T.: Will you accept the enclosed 10s. as a small donation to the Hospital Convalescent Fund, which I send you with great pleasure? I am sure all nurses must feel very grateful for such an excellent fund being in existence and be glad to help in ever so small a way.

### "THE DEARTH OF TRAINED NURSES."

"NURSE M. E. A.," Dublin, writes: I have been for many years a trained nurse in connection with the City of Dublin Nursing Institution, and during that time cannot speak too highly of the kindness I have received, both from the directors and the lady manager. We have at all times found in Mrs. Kildare-Treacy a true and kind friend, ever ready to help and advise us, and administer to our comforts. The home is very comfortable, the food extremely good, and the discipline such as to guide us through the quicksands of our profession. We are kindly and carefully looked after when ill, and liberally treated as regards holidays. No one I know has ever had just cause for complaint against this institution, which numbers at least 130 trained nurses on its staff.

"B," in thanking "Bluebell" for having started a discussion on the subject, adds: There is one more suggestion I would like to mention. No thoroughly trained high principled nurses will join institutions whilst they are restricted regarding religious opinions. I wrote to an old-established institution not long since. The answer came that I must be Church of England. Surely, a Christian nurse ought to be allowed to go where she wishes. We are in England a free country. Then again, "A Lady Superintendent" is greatly mistaken as regards small institutions. The matron in a small house knows her nurses better and selects the most suitable, not in rotation, as "Lady Superintendent" suggests. But it is true that large institutions are very unhomelike, and small consideration is often shown for the comfort of the nurses.

"AN OLD PRIVATE NURSE" writes: Thinking I should like to join a private nursing home in London, and seeing one advertised in THE HOSPITAL which I thought would suit me, I called one morning to see the matron. I found she was not at home, but the assistant saw me. I mentioned my previous experiences, and she gave me a few particulars and informed me that the salary was £25 a-year, with full in and out door uniform. She was certain the matron would engage me, but could not say definitely, and asked me to call again. I did so a few days after, and saw the matron, who politely informed me that she did not think much of infirmary nurses, and that she never took any notice of a nurse's testimonials, "for they were only a farce"—that was her expression. She also said that the salary was £20, not £25, as stated. Needless to add, I declined her offer to go for a month on trial. In my opinion no nurses of three years' training should take up private work in homes of this class. Minute inquiries ought always to be made.

### THE METROPOLITAN ASYLUMS BOARD HOSPITALS.

"A CONSTANT READER" writes on this subject: I read with pleasure "An Old Nurse's" letter in "The Mirror." As there seems to be such strong aversion on the part of most trained nurses to enter a fever hospital, I should like to add my experience to that of "An Old Nurse," and say that I found the work most interesting, and that I also, during the two and a half years I spent as charge nurse under the Metropolitan Asylums Board, learnt a good many things not possible to acquire in a general hospital, which have proved of great service to me since, both abroad and in England. The medical superintendent and matron were always ready to aid the nurses in any matter which tended to the greater comfort or amusement of the patients or of the nurses themselves; and I found the medical staff were at all times willing to give any information regarding the cases or their treatment of them. We also attended lectures on artificial feeding and infectious work generally, which were most instructive.

"K. M." also writes: It gave me great pleasure to read the letter of "An Old Nurse" about the North-Eastern Hospital. May I also, as another old nurse of the Metropolitan Asylums Board, say a little about my experiences in one of their hospitals? When I talked of going there to some of my nurse friends they said I should not like it, and spoke rather disparagingly of the class of nurses employed. However, I went as charge nurse to the North-Western Hospital, Hampstead, and spent sixteen very happy months there. I learned many useful things which I never could have learned in a general hospital. The rooms and food were excellent, the off-duty time exceptionally good, and the nurses (with the always inevitable few exceptions) sociable and kind. There are a great many admirable appointments in fever hospitals, and nurses who have had a practical training in infectious diseases are usually preferred. Nurses looking for promotion and also private nurses would find it a great benefit to spend at least one year in one of our Metropolitan Asylums Board's hospitals. I consider that a nurse's training is incomplete until she has had practical experience in the nursing of infectious diseases.

### ARE NURSES EXTRAVAGANT?

"ONE WHO WORKS ON THE CO-OPERATIVE PRINCIPLE" writes: It is a pity that your correspondent, "A Nurse of Twelve Years' Experience," cannot be Chancellor of the Exchequer. She would then be able to direct the public purse instead of that of her fellow nurses. My experience in private nursing on the co-operative principle extends considerably over twelve years, and I have mixed with all sorts and conditions of nurses, but I must say I never met with one of the type she describes. Your correspondent may feel elated at having six pounds in her purse, but it is a very usual thing for a nurse working on the co-operative principle to have twice that sum. But when she considers the uncertainty of her next case, and that she has to pay for board and lodging, &c., she does not need to go to the Holborn and to theatres to spend her money. As for going to Brighton or other seaside place between her cases, I think the nurse who does it is wise, for she can live as cheaply there as in London, and she brings back a fresh stock of strength and vigour to her next patient. With regard to the last remark, about nurses betting on races, all true nurses must ignore it as entirely beneath their notice.

### THE POSITION OF NURSES UNDER THE POOR LAW.

"PHYLLIS" writes: I do not see what we shall gain by appealing to the Local Government Board. They can only make orders which take years to come into general use. Would it not be better to form an association of ourselves? Two or three might meet locally and discuss privately what they are trying to effect in their several infirmaries, what is possible or advisable to attempt, thus building up a code of etiquette which as a new body we should need. I think it is clear that the intention of the order of 1897 is to provide a really good nurse where she is most needed, viz., where the medical officer is not resident, thus relieving the matron of the responsibility of the nursing, so that no superintendent nurse should professionally be under her rule. If these superintendent nurses then devote themselves to nursing proper, letting all other matters go the way of the House, they will save themselves much worry. Their charge nurses should be directly answerable to the master and matron for stores, linen, cleaning. The tendency all over the country is to provide separate establishments for the children, sick imbeciles, and now the aged, but till this arrangement becomes general let us make the best of the order, being grateful for the official recognition of the value of three years' training, and also for the plain language in which pauper nursing is forbidden, and which it would appear to be our special mission to carry out.

### The International Congress of Women.

The official programme of this Congress is rather an overwhelming document. No less than sixty meetings have been arranged for, and as many as five will take place simultaneously. The head-quarters of the Congress are to be at Westminster Town Hall, the Convocation Hall of the Church House and St. Martin's Town Hall being also requisitioned for sectional meetings. On the opening day, Monday, June 26th, the International Council will receive and welcome their guests at Westminster Town Hall, the Countess of Aberdeen will deliver her presidential address, and the same evening a reception will be held at Stafford House. On Tuesday business begins, educational matters coming first on the programme. In this section is included papers on the training of mentally and physically defective children, and it is satisfactory to note that Dr. Warner and Mrs. Burgwin, whose names are so closely associated with this subject in England, are to be among the speakers. The political enfranchisement of women, of course, will occupy a chief place in the political section, and it will be particularly interesting to many women to hear Mrs. Charlotte Stetson, the author of "Women and Economics," speak on the "Ethics of Wage Earning." Those who are interested in prison reform will be glad to be reminded that Mrs. Ellen C. Johnson, superintendent of the Reformatory Prison for Women at Boston, will explain the more enlightened methods of America which many would like to see carried out in this country.

### Minor Appointments.

Stepney Union Infirmary, Bromley-by-Bow.—On March 16th Miss Elizabeth S. Owen was appointed Superintendent Nurse. She was trained at the Nurses' Training School, Royal Infirmary, Liverpool; she has been charge-nurse at Toxteth Park Hospital, Liverpool, and at Fir Vale Infirmary, Sheffield; and superintendent nurse at the Union Infirmary, Luton.

Belper Joint Hospital.—On May 16th Miss L. Martin was appointed Matron. She trained at the Addenbrooke's Hospital, Cambridge. Her subsequent appointments were at the City of Liverpool Hospital for Infectious Diseases; the Victoria Home, Cheltenham, for Midwifery; and she has had considerable experience in private and district nursing.

Miss Caroline E. Dowker has been appointed Matron of the General Hospital, Ramsgate. Miss Dowker was trained at the Royal National Hospital, Margate, where she held the appointment of sister. Subsequently she held the appointment of matron of Wanstead Orphanage, Margate, and she has also had seven years of private nursing.

Middlesborough Union.—Miss M. E. Marshall was appointed Superintendent Nurse of the Workhouse Infirmary on May 18th. She was trained at the Cardiff Union Workhouse Hospital. She has been harge nurse at Cardiff Union, and the Union Hospital, Liverpool.

The Hospital, Altrincham.—Miss C. Symonds has been appointed Night Superintendent. She was trained at St. Bartholomew's Hospital, Rochester, and has been for the last two years ward sister of the Brentford Union Infirmary, Isleworth, W.

Brentford Union Infirmary, Isleworth, Middlesex.—Miss Helen Raggett was appointed Ward Sister on May 17th. She was trained at St. Saviour's Infirmary, East Dulwich.

### For Reading to the Sick.

"My son, I am the Lord who gives thee strength in the day of the trouble. Come to Me, when it is not well with thee."

"Wait for Me; and I will come and heal thee."

God sends sometimes a stillness in our life—
The bivouac, the sleep;
When on the silent battlefield the strife
Is hushed in slumber deep;
When wearied hearts exhausted sink to rest,
Remembering nor the struggle, nor the quest—
He giveth rest, more perfect, pure and true,
While we His burden bear.
It springeth not from parted pain, but through
The accepted blessing there;
The lesson, pondered o'er with thoughtful eyes,
The faith that sees in all a meaning wise.

*L. Fletcher.*

"Doth God indeed know my pain?" the weary spirit cries; can He understand our weakness or comprehend how his judgments affect our finite minds? We know that He can.

In our Blessed Lord himself we behold God sharing the experience of human frailty, sharing our weakness, our limited powers.

He Himself knows what it is to be bound by the flesh, to be in chains through physical inability to rise, to be unable to cast off the daily details of surroundings, or to be free from the cares of the body. And not only knows, but He cares for us; and, which is more than we can ever really know, understands how to lead us to the best completion of our powers, the most perfect fulfilment of our being.

We must be sure not only that He "knoweth," and that he careth, but also that His knowledge and His care place us in the very best circumstances, and with the most perfect possibilities of happiness and growth that could ever be ours. He cultivates His saints. He tends His children as the master gardener his most cherished plants, and He understands their needs.

He knows all our virtues, our faults, our weakness, and our strength; and He is faithful, and will bring us to the fulness of our life in Him if we will only trust him wholly with our lives. —*E. A. D.*

If Himself He come to thee, and stand
Beside thee, gazing down on thee with eyes
That smile and suffer; that will smite thy heart
With their own pity to a passionate peace;
And reach to thee Himself the Holy Cup,
Pallid and royal, saying "Drink with Me!"
Wilt thou refuse? Nay, not for Paradise!
The pale brow will compel thee, the pure hands
Will minister unto thee; thou shalt take
Of this Communion through the solemn depths
Of the dark waters of thine agony,
With heart that praises Him, that yearns to Him
The closer from that hour. Hold fast His hand
Though the nails pierce thine too! Take only care
Lest one drop of the sacramental wine
Be spilled, of that which ever shall invite
Thee, soul and body, to thy living Lord.—*H. H. King.*

My God, help me to obey Thy word, and be still; quiet my heart, teach me my own littleness and nothingness, give me grace to leave all to Thee. Nothing can go wrong which Thou dost order; nothing can suffer by my being laid aside if Thou Thyself dost take it in hand. Give me humble resignation; work in me by Thy Spirit a perfect willingness to lie still as long as it is Thy will that I should, and lead me to rest tranquilly in Thee. —*Bourdillon.*

In the weary hours of sickness,
In the times of grief and pain,
When we feel our mortal weakness,
When the creature's help is vain,
By Thy mercy,
O deliver us, good Lord. —*Hymns A. and M.*

### Travel Notes.

By Our Travelling Correspondent.

### XXIV.—FLORENCE AS AN INTERMEDIATE STATION.

Florence cannot in any way be considered a health resort, though I shall have something to say even on that matter presently, but it is so convenient and suitable as a place of call between the extreme warmth of the south and the breezy coolness of the mountains that it is worthy our consideration for that reason alone.

### The Journey.

From London first-class *viâ* Dover, Calais, Paris, and the St. Gothard, £9 3s. 10d.; second-class, £6 9s. A cheaper route is by Newhaven and Dieppe, first-class, £7 16s.; second, £5 10s. 3d. You may also go *viâ* Calais and Laon, not touching Paris at all; first-class, £9; second-class, £6 6s. 4d. This latter way is good for those who are sufficiently robust to go through to Milan without stopping, which is reached on the evening of the second day. There are altogether a choice of some twenty-five different routes by which to reach Florence; the expense is about the same by all lines.

### The Best Season for Florence.

To my partial mind all seasons are alike delightful in Florence, but for those who are in any way delicate spring and autumn, but more especially spring, is the ideal season. Supposing that an invalid has been wintering in Egypt or Sicily or Amalfi, or indeed any sheltered spot, and proposes to spend the summer in the Tyrol or in Switzerland, I think no place is better to choose than Florence for between seasons —it is so brilliantly sunny, so full of interests of all kinds, and so conveniently placed for easy excursions. Something of the same sort may be said of Venice, which is full of charms of its own. Spring in Venice is a thing to remember all one's life, but for the majority I think Florence is more suitable. The healthy can enjoy the winter in this beautiful city of lilies, but it is intensely cold; north-east winds sweep through the streets with a cruel force, and the brilliancy and heat of the sun seem to accentuate their ferocity, but when March and April come it is absolute perfection, and you may remain there till the middle of June, after which date it will be wiser to seek higher latitudes, somewhere in the Tyrol or in Switzerland.

### Accommodation, Hotels, &c.

Even those with very slender purses may live in Florence if they do not mind humble surroundings. One friend of mine spent the entire autumn in a little pension, where he paid only 4 frs. a day, and assured me that he was quite comfortable. The principal hotels charge about 15 frs. per day. Those situated on the Lungarno are flooded with sunshine, if you choose rooms looking to the river. Apartments are good and fairly moderate in Florence. You can get two or three rooms for 65 frs. a month, but some of the more luxurious rooms are highly rented. There are, I should say, some hundreds of pensions in Florence, from the most modest prices up to such establishments as Piccioli's in the Via Tornabuoni, or the Chapman in the Via Pondolfini.

### English Churches and Library.

Of churches there is no lack. Two Anglican, one American, one Presbyterian, and one Roman Catholic supply all needs, and the English and American shifting population of Florence is so large that all four are full. Vieussieux's circulating library is one of the best, if not quite the best, on the Continent, and the terms are most liberal; you may even subscribe for a single week, and the owners are the most accommodating and unsuspicious people possible, allowing you to take away the books on tour and to change them through the post. They have almost all the new standard works, and many copies of each, and their polite and obliging ways make a visit to their splendid library always a pleasure.

### Housekeeping in Florence.

Living may be very reasonable in Florence for those who are resident and who understand Italian, otherwise I recommend hotels and pensions. Meat and fish are dear, and coffee and tea also, but, to balance this, wine, fruit, vegetables, &c., are extremely cheap. My housekeeping cares have been generally limited to getting milk for afternoon tea, which is achieved in a novel manner. The impoverished Italian

The Troce del Frebbio.

nobility send up milk, butter, and eggs from the country farms where they live to their Palazzi in Florence. These are usually let, but they reserve to themselves the cellar floor, and here you go and purchase one sou's worth of milk, which is supplied in a curiously-shaped glass bottle; this you return the next day on going for your next supply. I patronised an aristocratic stall of this sort in the basement of a grand old palace near the Strozzi, and found the milk and butter excellent. The Italians are a simple race, and see no humiliation in openly selling their produce if they need to do so. They still remain dignified and stately and are quite willing that the "Forestieri" should clamour round their magnificent 15th century portals for ha'porths of milk and screws of butter. They have none of our crude self-consciousness and ever retain their air of grand signors under the most trying circumstances. There are English tea-rooms opened lately in the Lungarno Acciavoli and at the Albion.

This is a great convenience if you want to have several friends to tea and are too much pressed for time to visit the noble milksellers.

AMUSEMENTS, CABS, AND TRAMS, theatres, concerts, &c., are cheap—far more so than in the south of France—and the means of going to them and of getting about generally are very reasonable. Cabs 1 fr. the course, at night 1 fr. 30 c. Omnibuses and trams abound. For a penny you can go as far as any of the city gates from the Piazza della Signoria. Trams traverse the city in all directions, and now one can go in great luxury to beautiful Fiesole in the electric tram for the magnificent sum of 70 c. and to the Certosa for 40 c.

DOCTORS, NURSES, CLUBS, &C.

There are numerous English doctors, and excellent dentists, and of late years more than one nursing institute has been started. This latter improvement was greatly needed, and has been much appreciated. There is also a small hospital to which all are admitted, irrespective of nationality, on the very modest payment of 12 frs. a day. There are four clubs. The Florence, in the Piazza Vittorio Emanuele, is well appointed—six months' subscription, 100 frs. There is also a lawn tennis club (nominations by members), asphalte and gravel courts. This is out by the Cascine. The shops are excellent, and it is quite possible (if you do not mind paying for the luxury) to have English groceries and English tailoring. Tea is very dear, and when made according to the original conception of the Italians it is an obnoxious brew. They keep it in cardboard boxes, and consider one meagre spoonful floating in an ocean of hot water "excellent for the nerves." They invariably make it in a china teapot of generous dimensions, which has never been in the region of a stove, and serve the straw-coloured lukewarm beverage with a proud air, as one who should say "we know perfectly the tastes of these English." The making of coffee they do understand, but from its high price they are not unnaturally inclined to stint the supply, and I have yet to learn that good coffee can be made without a sufficiency of the berry itself. Next week we will consider the attractions of Florence for the spring visitor.

---

### TRAVEL NOTES AND QUERIES.

ST. SERVAN (Linguist).—The roads about St. Servan, Dinard, and Dinan are excellent for cycling, and but little funds will carry you through. Out of the towns they speak no English, but are so quick and intelligent, so polite and anxious to help that you will find no difficulty. Madame Pallot, Maison Mazzias, will suit you, 7 frs. per day; possibly less by arrangement if not in the season. Write to her and ask. Miss Humphreys, Rue de Pomelle, is also reasonable, and Miss Dixon, Place Constentive. Seven francs is just within 40s. a-week. There is nothing cheaper in St. Servan. In Paremé they profess to take you at the Hôtel des Bains and Hôtel de France for 6 frs. per day, but then there will be more tipping, which brings it practically to 7 frs. a-day.

KNOCKE (Belgium).—I cannot remember your pseudonym, but I thank you for your interesting notes on Knocke. I know it well, but am always very glad to have independent information. It is all you say and a miracle of cheapness.

INNSBRUCK FOR WINTER (Alba).—Yes, it has become quite a winter resort for the English of late years. The extreme dryness of the atmosphere makes the cold hardly noticeable. Living is reasonable. Hotel Tirol inexpensive and very comfortable. I think the proprietor will meet you as to terms. Cost of the journey, first-class return, £9 3s. 5d.; second ditto, £6 10s. 2d.

ROME FOR WINTER (Elsa).—It is delightful, but you must quite understand that it is by no means warm. The larger allowance of sun makes it much pleasanter than England, but on the other hand, the Romans do not understand warming their houses as we do, and seem to consider the sun's rays the only legitimate producer of warmth. Plenty of warm wraps and unlimited wood fires are necessary to the enjoyment of a Roman winter. Apartments are not dear compared to the Riviera, but if you only stay three months it is less trouble to arrange at a moderate hotel or pension.

OPEN AIR TREATMENT IN DAVOS PLATZ (Hope).—I fear I cannot encourage you much in your idea. I think you would stand no chance of obtaining such work as you require. The doctor who sent out the patient would probably recommend a nurse if he was sufficiently ill to need one. This matter, however, hardly comes under my department, and if you like you might ask in "Notes and Queries" on the last page as to your chances of success. With respect to the expenses of the journey, first-class single is £5 12s. 6d.; second-class, £3 19s. Returns are only for 45 days, and therefore not available. The treatment varies in the length, therefore I hardly know how to tell you. Board and lodging without doctors' fees, use of sanatorium, or any extras whatever would be from £20 to £25 for two months, reckoning pension terms at eight francs per day. For clothing take woollen only, both upper and under; let it be light but warm, with silk blouses lined with thin flannel to wear in the evening.

*(For Travel Advertisements see Page* xviii.*)*

### Notes and Queries.

**The contents of the Editor's Letter-box have now reached such unwieldy proportions that it has become necessary to establish a hard and fast rule regarding Answers to Correspondents. In future, all questions requiring replies will continue to be answered in this column without any fee. If an answer is required by letter, a fee of half-a-crown must be enclosed with the note containing the enquiry. We are always pleased to help our numerous correspondents to the fullest extent, and we can trust them to sympathise in the overwhelming amount of writing which makes the new rules a necessity.**

**Every communication must be accompanied by the writer's name and address, otherwise it will receive no attention.**

---

*Training.*

(78) I wish to obtain a post as probationer in some hospital or infirmary, but have not had a good education. Kindly tell me if that is necessary in every case?—*R. S. W.*

"A good education" is made of more importance as a qualification at some institutions than at others. Apply at some of the Poor Law training schools, of which you will find full particulars in "The Nursing Profession," published by the Scientific Press, 28, Southampton Street, Strand, W.C.

*Turkish Baths.*

(79) Would some one kindly recommend me where to go for a Turkish bath?—*A. M. H. (London).*

Turkish baths for ladies are provided at most of the larger public baths in London, and are advertised generally.

*Au Pair.*

Could you kindly inform me if there is any hospital or institution where I could receive training in monthly nursing in return for services?—*Laura.*

A few private homes are open to arrangements of this kind, and a short advertisement would probably bring several replies. You must, however, be careful to investigate references before engaging yourself, so as to make sure that you would obtain the training you require.

*Monthly Nursing Abroad.*

(81) Can you inform me how to obtain information of German or Continental hospitals where I could obtain a short training in general work, or take a post as monthly nurse?—*Mary L.*

We cannot take the responsibility of recommending foreign institutions. The conditions of service are so different that, unless private reasons render the emigration desirable, it is much better for trained nurses to stay at home, unless they have proper introductions.

*Nursing Directory.*

(82) "E. D." would be very glad to know how she could get her name put in the Nurses' Directory, and to whom must she apply?

The Editor of "Burdett's Official Nursing Directory," 28 & 29, Southampton Street, Strand, has always much pleasure in receiving authentic names and details of certificated nurses.

*R. B. N. A.*

(83) Would you kindly tell me if I could be a member of the Royal British Nurses' Association? I have had three years' general work in a good hospital, and 15 months' fever training. Where should I write for rules, &c.?—*D. J. B.*

By applying to the secretary, at 17, Old Cavendish Street, W., you will be furnished with all particulars.

*Hospital Refuse.*

(84) I should be glad if you would inform me of the best method for disposing of the hospital refuse, ashes, dressings, &c., in a small hospital.—*Secretary.*

All dressings, &c., ought to be destroyed by fire. The ashes are frequently placed in large pails or small sanitary bins that can be carried and emptied directly into the dust-cart.

*Paralysis.*

(85) I should be obliged if you could give me any information as to homes or hospitals for those suffering from paralysis. I am asking on account of a gentleman who for some time past has been gradually losing the use of his limbs, and as he is growing rapidly worse his friends fear that it is a case of incurable paralysis. They are not in a position to support him entirely, and wish to know of any homes for such cases where patients can be received for a moderate sum.—*B. R. H.*

You will find a list of hospitals and public homes for such cases under the index title "Paralysis" in Burdett's Hospitals and Charities," price 5s., published by the Scientific Press, London, W.C.

*Bicycle Baskets.*

(86) Can you tell me if there are any baskets made specially for a nurse's bicycle; if so, where obtained?—*T. B. T.*

Any of the large emporiums would be able to get you one, as, if they are not kept in stock, one could easily be made to order. Southall Bros. make a nice one.

*Home for Child.*

(87) I should be very much obliged if you could tell me of any institution which would receive a healthy female child three months old. Mother, labourer's wife, a violent epileptic. Five shillings a week could be paid, also reliable references could be given.—*Enigma.*

We cannot recommend any institution of the kind, but in the "Englishwoman's Year Book" (price 1s.), published by F. Kirby, 17, Bouverie Street, Fleet Street, E.C., you will find [illegible] ery complete list of such homes.

## Figures and Tables

**Figure f1:**